# The Case for Implementation Science in Medical Radiation Practice

**DOI:** 10.1002/jmrs.70072

**Published:** 2026-02-26

**Authors:** Laura Di Michele, Mitchell Sarkies, Nicola Creagh

**Affiliations:** ^1^ School of Health Sciences, Faculty of Medicine and Health The University of Sydney Sydney New South Wales Australia; ^2^ Implementation Science Academy Sydney Health Partners Sydney New South Wales Australia; ^3^ Melbourne School of Population and Global Health, Faculty of Medicine, Dentistry and Health The University of Melbourne Melbourne Victoria Australia

## Abstract

Initiating and sustaining clinical change can be incredibly challenging. Implementation science offers methods, tools and frameworks to systematically drive the uptake of evidence‐based practices, speeding uptake and driving sustained practice change. The fields of Medical Radiation Science would benefit significantly by utilising the knowledge base from the discipline of implementation science and translating this within our context to realise clinical change more effectively and contribute our own specific knowledge to this field.

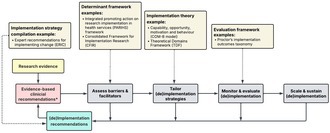

Medical radiation science (MRS) is a rapidly evolving field with high stakes. The stakes are particularly elevated in paediatric populations where the potential for stochastic effects of radiation is more pronounced [1, 2], and psychological risks such as anxiety and distress are cause for pause for Medical Radiation Scientists collectively [3]. There is an expanding body of empirical research, both in paediatric and broader MRS settings; however, when this research is not effectively translated into practice change in clinical settings, we risk harm to our patients.

In this issue, Ryan and colleagues provided an exemplar application of implementation science to realign medical imaging practice to updated guidance on patient contact shielding [4]. They describe a structured implementation approach to the discontinuation of patient contact shielding in a paediatric institution [4]. This serves as an excellent demonstration of an ongoing journey towards evidence implementation in radiography.

The discontinuation of patient contact shielding in paediatric patients may appear, on the surface, to be relatively straightforward; it has no cost implications and is within the scope of radiographers to implement without input or approval from radiologists. The discontinuation of shielding has excellent evidence; however, despite this, it has been a contentious debate among radiographers since the AAPM Position Statement on the Use of Patient Gonadal and Fetal Shielding in 2019 [5]. Internationally, the use of contact shielding remains relatively frequent, with a recent European survey reporting that while 70% of institutions had formally introduced a no‐shield policy, 74% of radiographers would still shield paediatric patients regardless [6]. What sets Ryan and colleagues' study apart from the pack is the use of a structured implementation science approach.

Implementation science, as a methodological discipline, provides the tools and conceptual frameworks to systematically drive the translation of research evidence into routine practice [7]. This includes both the adoption of new practices based on emerging evidence, as well as the discontinuation of practices where subsequent evidence no longer supports their use (de‐implementation), with the ultimate goal to improve the quality of healthcare and optimise health outcomes [8]. The tools and conceptual frameworks of implementation science allow for: (a) identifying contextual barriers that prevent the uptake of new practices, or the persistence of low‐value care; (b) selecting implementation strategies that address these contextual barriers; and (c) monitoring and evaluating implementation efforts to assess the evidence‐to‐practice alignment. By turning to the literature and utilising this to inform our choices of strategies to support implementation, the likelihood of successful practice change is increased, allowing resources to be allocated more strategically [9].

By utilising the Capability, Opportunity, Motivation and Behaviour (COM‐B) model [10] and Theoretical Domains Framework (TDF) [11] in conjunction, Ryan and colleagues were able to systematically identify barriers to implementation and develop strategies to mitigate these with great success. Relying on patient and provider education strategies alone may not have achieved the remarkable outcomes reported in this study. Barriers to implementation were identified across several behaviour change domains, which are often overlooked, such as the environment and choice architecture (i.e., removing easy access to shielding), beliefs and perceptions about shielding, and behavioural management through senior staff modelling and clear protocols. Implementation science theories, models and frameworks aim to guide the strategic selection of these activities to support the adoption, scale‐up and sustainability of evidence into policy and practice. They serve different purposes, such as describing the process of translating research into practice, understanding what factors influence implementation success, and evaluating implementation [12]. While the choice of the COM‐B and TDF suited the implementation challenges faced by Ryan and colleagues, ultimately, the choice of framework should align with the unique needs of each implementation project.

Whilst the field of implementation science is well established, the application and reporting of implementation principles among MRS remains in its infancy [13]. Whilst there are some excellent examples of the application of implementation science principles in MRS [14, 15, 16], this remains an area in which there is significant potential for contextualised application and dissemination which will advance the profession. For medical radiation professionals seeking to incorporate implementation science into their practice improvement efforts, conceptual models, such as the model by Sarkies and Jones et al. provide practical guidance for applying implementation science into the translation of evidence into practice (Figure 1) [17]. This model proposes integrating implementation science methods throughout the evidence translation process, which involves engaging stakeholders, identifying barriers and facilitators, and selecting strategies informed by theory; all of which was exemplified by Ryan and colleagues. The systematic application of such models offers ample opportunity to improve clinical practice in MRS, both within and across settings.

**FIGURE 1:**
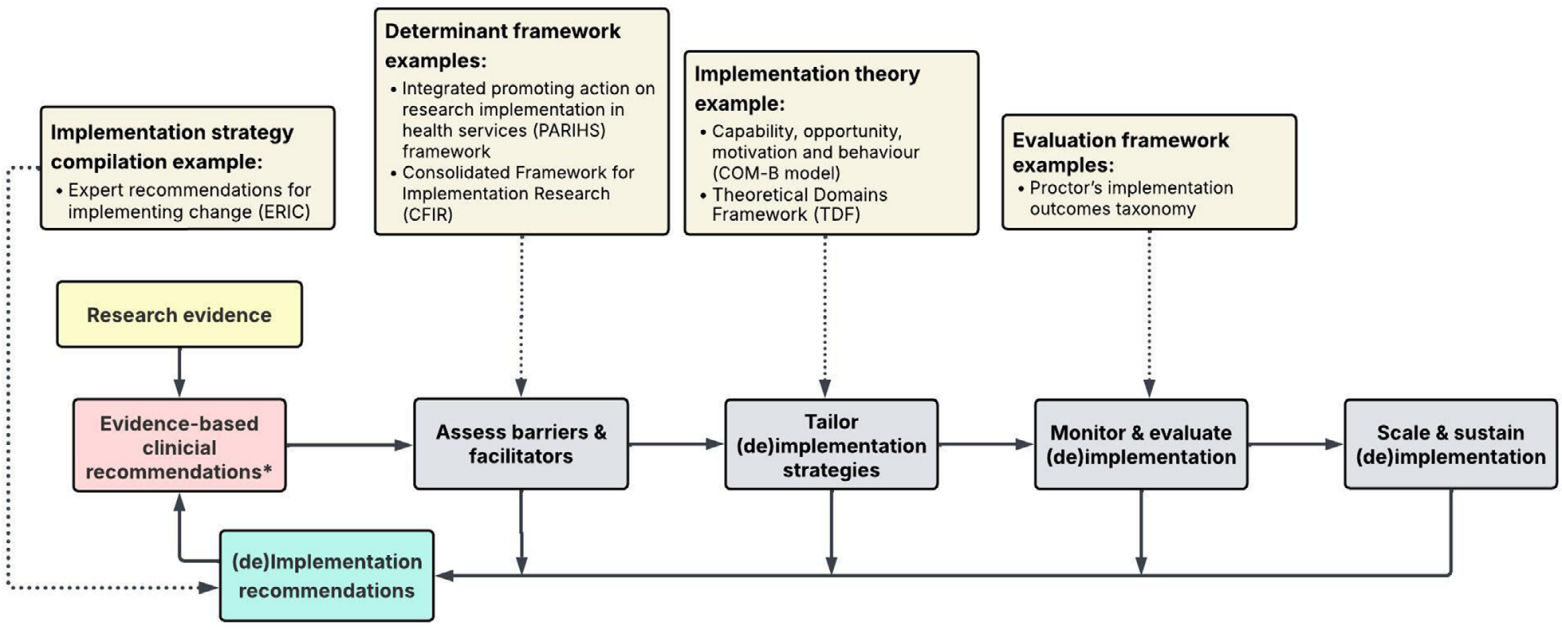
Conceptual model of the integration of implementation science in translation of evidence into practice, adapted from Sarkies et al. [17]. *Evidence‐based clinical recommendations include interventions, such as the discontinuation of shielding in paediatric patients.

In the fields of MRS, the evolution of practice is often dictated by technological advancement [18], however this technological advancement does not always equate to timely improvements in patient care [19]. Professional culture often compounds these challenges, with limited motivation, poor organisational culture and medical dominance often cited as barriers to evidence‐based practice by radiographers [13, 20]. Indeed, changing ingrained practice is hard, particularly when it involves unlearning old evidence [21].

Progress in medical radiation science depends on our willingness to consistently question ways of working and reimagine care for the better. The 2026 Professional Capabilities for Medical Radiation Practitioners require that medical radiation scientists are not only lifelong learners, but leaders and stewards, working towards “efficient, effective operation and continual improvement of the healthcare system” [22]. As we progress towards this improvement, implementation science provides us with the tools and strategies that we need to realise that change efficiently and evaluate its effectiveness.

## Conflicts of Interest

The authors declare no conflicts of interest.

## Data Availability

Data sharing not applicable to this article as no datasets were generated or analysed during the current study.
